# “Working the System”—British American Tobacco's Influence on the European Union Treaty and Its Implications for Policy: An Analysis of Internal Tobacco Industry Documents

**DOI:** 10.1371/journal.pmed.1000202

**Published:** 2010-01-12

**Authors:** Katherine E. Smith, Gary Fooks, Jeff Collin, Heide Weishaar, Sema Mandal, Anna B. Gilmore

**Affiliations:** 1 School for Health, University of Bath, Bath, United Kingdom; 2 Centre for International Public Health Policy, University of Edinburgh, Edinburgh, United Kingdom; 3 London School of Hygiene and Tropical Medicine, London, United Kingdom; University of California San Francisco, United States of America

## Abstract

**Background:**

Impact assessment (IA) of all major European Union (EU) policies is now mandatory. The form of IA used has been criticised for favouring corporate interests by overemphasising economic impacts and failing to adequately assess health impacts. Our study sought to assess how, why, and in what ways corporations, and particularly the tobacco industry, influenced the EU's approach to IA.

**Methods and Findings:**

In order to identify whether industry played a role in promoting this system of IA within the EU, we analysed internal documents from British American Tobacco (BAT) that were disclosed following a series of litigation cases in the United States. We combined this analysis with one of related literature and interviews with key informants. Our analysis demonstrates that from 1995 onwards BAT actively worked with other corporate actors to successfully promote a business-oriented form of IA that favoured large corporations. It appears that BAT favoured this form of IA because it could advance the company's European interests by establishing ground rules for policymaking that would: (i) provide an economic framework for evaluating all policy decisions, implicitly prioritising costs to businesses; (ii) secure early corporate involvement in policy discussions; (iii) bestow the corporate sector with a long-term advantage over other actors by increasing policymakers' dependence on information they supplied; and (iv) provide businesses with a persuasive means of challenging potential and existing legislation. The data reveal that an ensuing lobbying campaign, largely driven by BAT, helped secure binding changes to the EU Treaty via the Treaty of Amsterdam that required EU policymakers to minimise legislative burdens on businesses. Efforts subsequently focused on ensuring that these Treaty changes were translated into the application of a business orientated form of IA (cost–benefit analysis [CBA]) within EU policymaking procedures. Both the tobacco and chemical industries have since employed IA in apparent attempts to undermine key aspects of European policies designed to protect public health.

**Conclusions:**

Our findings suggest that BAT and its corporate allies have fundamentally altered the way in which all EU policy is made by making a business-oriented form of IA mandatory. This increases the likelihood that the EU will produce policies that advance the interests of major corporations, including those that produce products damaging to health, rather than in the interests of its citizens. Given that the public health community, focusing on health IA, has largely welcomed the increasing policy interest in IA, this suggests that urgent consideration is required of the ways in which IA can be employed to undermine, as well as support, effective public health policies.

*Please see later in the article for the Editors' Summary*

## Abstract 2

Katherine Smith and colleagues investigate the ways in which British American Tobacco influenced the European Union Treaty so that new EU policies advance the interests of major corporations, including those that produce products damaging to health.

## Editors' Summary

**Background:**

The primary goal of public health, the branch of medicine concerned with the health of communities, is to improve lives by preventing disease. Public-health groups do this by assessing and monitoring the health of communities, by ensuring that populations have access to appropriate and cost-effective health care, and by helping to formulate public policies that safeguard human health. Until recently, most of the world's major public-health concerns related to infectious diseases. Nowadays, however, many major public-health concerns are linked to the goods made and marketed by large corporations such as fast food, alcohol, tobacco, and chemicals. In Europe, these corporations are regulated by policies drawn up both by member states and by the European Commission, the executive organ of the European Union (EU; an economic and political partnership among 27 democratic European countries). Thus, for example, the tobacco industry, which is widely recognized as a driver of the smoking epidemic, is regulated by Europe-wide tobacco control policies and member state level policies.

**Why Was This Study Done?:**

Since 1997, the European Commission has been required by law to assess the economic, social (including health), and environmental consequences of new policy initiatives using a process called an “impact assessment” (IA). Because different types of IA examine the likely effects of policies on different aspects of daily life—a health impact assessment, for example, focuses on a policy's effect on health—the choice of IA can lead to different decisions being taken about new policies. Although the IA tool adopted by the European Commission aims to assess economic, environmental and social impacts, independent experts suggest this tool does not adequately assess health impacts. Instead, economic impacts receive the most attention, a situation that may favour the interests of large businesses. In this study, the researchers seek to identify how and why the EU's approach to IA developed. More specifically, the researchers analyze internal documents from British American Tobacco (BAT), which have been disclosed because of US litigation cases, to find out whether industry has played a role in promoting the EU's system of IA.

**What Did the Researchers Do and Find?:**

The researchers analyzed 714 BAT internal documents (identified by searching the Legacy Tobacco Documents Library, which contains more than 10 million internal tobacco company documents) that concerned attempts made by BAT to influence regulatory reforms in Europe. They also analyzed related literature from other sources (for example, academic publications) and interviewed 16 relevant people (including people who had worked at the European Commission). This analysis shows that from 1995, BAT worked with other businesses to promote European regulatory reforms (in particular, the establishment of a business-orientated form of IA) that favor large corporations. A lobbying campaign, initiated by BAT but involving a “policy network” of other companies, first helped to secure binding changes to the EU Treaty that require policymakers to minimize legislative burdens on businesses. The analysis shows that after achieving this goal, which BAT described as an “important victory,” further lobbying ensured that these treaty changes were translated into the implementation of a business-orientated form of IA within the EU. Both the tobacco industry and the chemical industry, the researchers argue, have since used the IA to delay and/or weaken EU legislation intended to protect public health.

**What Do These Findings Mean?:**

These findings suggest that BAT and its corporate allies have fundamentally altered the way in which EU policy is made by ensuring that all significant EU policy decisions have to be assessed using a business-orientated IA. As the authors note, this situation increases the likelihood that the EU will produce policies that favor big business rather than the health of its citizens. Furthermore, these findings suggest that by establishing a network of other industries to help in lobbying for EU Treaty changes, BAT was able to distance itself from the push to establish a business-orientated IA to the extent that Commission officials were unaware of the involvement of the tobacco industry in campaigns for IA. Thus, in future, to safeguard public health, policymakers and public-health groups must pay more attention to corporate efforts to shape decision-making processes. In addition, public-health groups must take account of the ways in which IA can be used to undermine as well as support effective public-health policies and they must collaborate more closely in their efforts to ensure effective national and international policy.

**Additional Information:**

Please access these Web sites via the online version of this summary at http://dx.doi.org/0.1371/journal.pmed.1000202.

- Wikipedia has a page on public health (note that Wikipedia is a free online encyclopedia that anyone can edit; available in several languages)
- More information on the European Union (in several languages), on public health in the European Union, and on impact assessment by the European Commission is available
- The Legacy Tobacco Documents Library is a public, searchable database of tobacco company internal documents detailing their advertising, manufacturing, marketing, sales, and scientific activities
- The World Health Organization provides information about the dangers of tobacco (in several languages)
- The Smoke Free Partnership contains more information about smoking prevalence in Europe and about European policies to tackle the public health issues associated with tobacco use
- For more information about tobacco industry influence on policy see the 2009 World Health Organization report on tobacco industry interference with tobacco control

## Introduction

Increasingly, many of the world's major public health concerns are linked to the goods produced and marketed by large corporations (such as those in the fast food, alcohol, tobacco, and chemical industries) [1]. Understanding how corporations influence policy therefore forms an essential part of public health research. Efforts to understand the behaviour of the tobacco industry, which is widely recognised as the vector of the tobacco epidemic [2]–[5], have been greatly facilitated by the release of internal corporate documents through a series of litigation cases in the United States. To date, research based on these documents has largely focused on industry efforts to influence tobacco control policy [6]–[14], and less attention has paid to what these documents reveal about collective efforts by corporations to influence broader policy debates. Our research sought to assess how, why, and in what ways corporations, particularly the tobacco industry, influenced the EU's approach to impact assessment (IA), by employing this unique resource. Policy theorists have long highlighted the importance of policy networks in achieving policy change [15]–[17], but there have been very few attempts (perhaps because of the difficulties in obtaining relevant data) to unpack how such networks are created. This paper offers some unique insights into the extent to which policy networks can be deliberately manufactured by sufficiently resourced actors and used to influence policy. It starts by introducing IA, the concerns around its use and its application within the EU.

### The Rise of IA as a Policymaking Tool

IA is used as a tool for evaluating potential legislative changes. Advocates claim it helps ensure that policy decisions are more transparent, rational [18]–[20], scientific [21], and democratic [20]. IA may incorporate a form of risk assessment (RA) (see Table 1) to assess whether the risks of a potential hazard are great enough to warrant regulation [22],[23]. Once a consensus has been reached about the need for action, the main IA tool is used to assess the potential impacts of different policy options as a way to inform decisions about the best way forward. Mirroring cost-benefit analysis (CBA), the methodology of IA is often understood to require all potential “costs” and “benefits” to be identified, converted into monetary units, and compared to determine the socially optimal policy option [24]. In practice, however, IA is often implemented more flexibly and does not necessarily involve the monetisation or quantification of all impacts [24],[25].

**Table 1:** The regulatory reforms BAT pursued.

Aspect of Regulatory Reform	Description	BAT's Interest
Risk assessment	A means of assessing the potential risk posed by a particular hazard. In a policy context, RA is designed to help inform decisions about whether legislative intervention is required to help manage a particular risk. It is thus usually undertaken early in the policymaking process.	From 1995 onwards, internal documents suggest senior managers at BAT believed a requirement for structured RA could be used to prevent legislation relating to ETS and tobacco advertising [268]. It recruited other companies involved in producing or marketing risky products to coalitions that worked together to attempt to redefine policymakers' approach to risk in the EU [62],[107].
Precautionary principle	The principle that, where there are reasonable grounds to believe that a given hazard would, if it occurred, result in severe or irreversible damage to the public's health or the environment, calls on policymakers to act to prevent that risk, even where there is not yet a scientific consensus about its likely occurrence and/or impact.	The companies involved in the above campaign were concerned about how the precautionary principle was being interpreted and employed by policymakers in the EU. Hence, in addition to trying to promote a form of RA that would work to businesses' advantage, they attempted to try to influence how this principle was understood and applied in the EU [107],[137].
Impact assessment	IAs are used as planning tools to inform and improve decision or policymaking processes. They usually aim to assess the likely effects of potential options in advance of their implementation. A variety of types of impact assessment exist, each focusing on different kinds of impacts, including ones relating to the environment, sustainability, inequality, health, social, and business interests.	BAT became interested in how IAs in Europe might be used to its advantage in the mid-1990s. By this point, a weak form of business IA had already been officially introduced in the European Community but had failed to significantly change the way in which policy was made [38],[269]. As this paper demonstrates, BAT subsequently recruited and contracted others to help it promote a requirement for IA that would work in its interests.
Business impact assessment	A form of IA that focuses on the impacts of potential policies on businesses. *Fiche d'Impact* was the first form of BIA introduced into the European Community in 1986 [270], but this system was limited and failed to significantly change the way in which European legislation developed [38],[269].	BIA is the form of IA that BAT was involved in promoting. The company perceived BIA to be a means of ensuring policymakers consulted with industry and took the potential costs of legislation to businesses into account when developing policy recommendations. In addition, it was seen as a way of pushing for structured risk assessment (see above) [87],[97],[100].
Cost-benefit analysis	A form of impact assessment that focuses on assessing potential costs and benefits of policies. Developed by economists, CBA usually requires costs and benefits to be quantified and converted into monetary terms [93],[251].	BAT largely used the term CBA, rather than IA, indicating that the kind of IA the company was interested in pursuing involved employing quantifiable (and, where possible, economic) impacts. Having been advised that qualitative benefits are often more difficult to assess than quantifiable costs [93], BAT was aware that a requirement to quantify might work to their advantage.
Stakeholder consultation	The basic idea that all those with an interest in a particular issue should be consulted. Interpretations of who constitutes a “stakeholder” vary. For example, the tobacco industry could be perceived as a key stakeholder in tobacco control policy or, in contrast, as a “vested interest.”	BAT appears to have been particularly keen to achieve a statutory requirement for policymakers to consult with potentially affected businesses at a very early stage in the policymaking process [62],[160]. This may be partly because tobacco companies were finding themselves increasingly excluded from policymaking discussions [271].

### Criticisms of IA

A large body of work critiquing the use of IA and CBA within policymaking (largely in the US) suggests that, rather than promoting transparency or rationality, these tools obfuscate political decisions concerning the prioritisation of interests in policy [26]–[28]. Various types of IA have been developed, including environmental impact assessment, sustainability impact assessment, health impact assessment (HIA), health equity impact assessment, and business impact assessment (BIA), each of which focuses on different kinds of impacts and is likely to lead to different conclusions about the best way forward. Hence, the decision to employ particular forms of IA and not others involves a political and ethical judgment about the criteria against which public policy should be assessed. Critics of IA/CBA suggest that this framing effect tends to privilege the interests of large corporations over other policy stakeholders (such as NGOs and public interest groups) in the following four ways. First, because it is easier to predict the more direct “costs” of regulations to business than the diffuse and long-term potential benefits for populations or the environment, costs to businesses may be over-represented [29]. Second, because much of the information policymakers need to assess impacts on companies is held by the companies concerned, there is a possibility that companies may share only the information that promotes their interests [30]–[32]. Third, the process of attaching monetised values to all predicted impacts (often regarded as necessary to facilitate the aggregation of dissimilar impacts) [20],[22],[33] is problematic because there is no agreed way of valuing some of the most fundamental impacts, such as lives saved [22],[28],[34],[35]. Fourth, IA provides a means for stakeholders to continually challenge, and thus delay, decisions with which they disagree [36].

### How Does IA Currently Operate in Europe?

Although the then European Community committed itself to employing a form of BIA known as *Fiche d'Impact* in 1986 [37], this was never fully embedded [38]. It is only since the 1997 Treaty of Amsterdam [39] provided a constitutional basis for IA [40] that IA has really begun to change policymaking in the EU and, in so doing, has put the European Commission, along with the UK, at the forefront of the drive for IA within Europe [41]. Since 2002, the European Commission (the main originator of legislation in the EU political process) has developed an approach to IA which ostensibly involves an “integrated” version of IA and aims to assess impacts in the three underlying areas (‘pillars’) of EU policy (economic, environmental, and social issues) [42]–[44]. The European Commission describes its IA tool as “a thorough and balanced appraisal of all impacts” [42]. However, independent assessments of the IAs it has produced claim that coverage across the three pillars is uneven, with economic impacts receiving the most attention [43],[44]. Health impacts (largely subsumed within the “social” pillar) have received particularly limited attention [45]–[47]; a review of the 137 IAs carried out by the European Commission in 2005 and 2006 found that more than half did not even mention the word “health” [45]. This is so despite the fact that Article 152 of the EU Treaty explicitly states that “a high level of human health protection shall be ensured in the definition and implementation of all Community policies and activities” [39], a statement that has been interpreted as requiring HIA for all EU policies [48]. Furthermore, it has been argued that IA in the EU is increasingly being interpreted as a means of achieving less, not necessarily better, regulation [44],[49]. The concerns raised by the reviews of the European Commission's recent IAs are consistent with a growing literature (summarised above) that suggests IA is most likely to benefit business.

### Objectives of the Study

Our analysis examines how, why, and in what ways corporations, particularly the tobacco industry, influenced the EU's approach to IA. We consider how IA has been, and is being, used by regulated industries in their attempts to influence EU policy. In order to explore these questions, we examined internal tobacco industry documents, combined with existing literature concerning IA and CBA, and undertook a series of interviews with relevant European informants.

## Methods

Following a series of litigation cases in the US, leading tobacco companies were required to make internal documents public [50]. These are now available online and can be searched using optical character recognition [51]. Their broad coverage means they also provide insights into the lobbying efforts of companies that worked with the tobacco industry, providing a unique resource for the analysis of corporate strategy and conduct.

We took an iterative approach to searching the tobacco documents, with broad initial search terms on tobacco industry attempts to influence European regulatory reform being used to generate more specific terms. Twenty-two initial search terms were used, including: “Better Regulation”; “cost-benefit” AND “eu*”; “Impact assessment” AND (“Eu*” OR “EC” OR “EP”); “risk assessment” AND “Eu*”; “sensible regulation” AND “Eu*”. Further specific search terms relating to particular texts, individuals, companies, lobbying and consultancy firms, trade organizations, networks, events, and places were then developed via the analyses of these documents. Some search terms were combined into search strings to minimize the number of documents returned in multiple searches.

We undertook our searches between mid-April and September 2008, initially using the BAT Document Archive (http://bat.library.ucsf.edu/) and, from July 2008, the newly integrated Legacy online library (http://www.legacy-library.ucsf.edu). The search strategy focused almost entirely on BAT internal documents, because preliminary searches of the internal documents of other tobacco companies indicated that it was the key tobacco corporation involved in the promotion of IA in Europe. In total, 5,677 documents were identified and reviewed. Where documents appeared particularly interesting or referred to other documents of interest, consecutive Bates numbers were searched. This method increased the total number of documents reviewed to just under 6,800. Of these, 714 were identified as relevant to BAT's attempts to influence regulatory reforms in Europe. These documents were imported to an EndNote library, from where they were re-read, interpreted, and thematically coded by an experienced qualitative researcher (KS).

The analysis of these documents was contextualised using secondary sources, including interviews with relevant individuals. Potential interviewees were identified via existing contacts known to the research team and other colleagues working in tobacco control, analyses of textual documents and recommendations by other interviewees. In total, 16 interviews were undertaken (four interviewees worked for European NGOs with an interest in public health, four were independent consultants or worked at relevant think tanks, and eight worked, or had previously worked, at the European Commission). After obtaining written consent, all interviews were digitally recorded and transcribed before being discussed within the research team and thematically analysed by an experienced qualitative researcher. In addition, electronic searches of the following sources was undertaken: Nexis (an electronic database of mass media publications); online databases of academic publications; Europa (the EU's Web site); Web sites of lobbying, consultancy, and trade organizations cited in the documents; accounts/transcripts of parliamentary debates (in the UK and EU); and public health and environmental NGO Web sites. This paper focuses primarily on the data produced by analysing BAT's internal documents but, by using the interview transcripts and the additional textual information to look for corroborating and conflicting evidence, we were able to offset some of the weaknesses inherent in document-based research [52],[53] and examine the ongoing impacts of the findings. Where the results described below are informed by interview data, this is specifically indicated; in all other cases, the results are based on an analysis of the cited documents. Interviewees are named only where consent was obtained to do so. The methodological approach was approved by the University of Bath's School for Health Research Ethics Committee.

## Results

### The Rationale Underlying the Push for Risk Assessment and IA

Following the failure of the *Fiche d'Impact* system to develop [54], in the mid-1990s BAT began to seriously consider lobbying for what it termed structured risk assessment (RA) within a framework of BIA/CBA [55]–[58]. Our analysis reveals that BAT saw RA as a means of precluding the introduction of public smoking restrictions [56],[59] which it saw as a growing threat in Europe [56],[60],[61]. It also appears that preventing tobacco advertising restrictions was thought to be another outcome [62]. BAT documents reveal that senior managers at the company had learnt that RA could be used in this way from US tobacco company Philip Morris [63]–[65], and its proposed strategy followed Philip Morris' campaign to redefine RA so that it could be used to challenge, rather than support, the classification of environmental tobacco smoke (ETS) as a group A (human) carcinogen [66]. BAT had links to two US legal firms, Shook Hardy and Bacon [63]–[65],[67]–[70] and Covington & Burling [71],[72], that had been involved in the US campaign [73]. Notably, Christopher Proctor, formerly a senior research scientist at BAT [74], had worked for Covington & Burling during this period [75]–[77] and, on return to BAT in 1994 [78],[79], was asked by a colleague (Stuart Chalfen, a solicitor at BAT [80]) to produce an overview of Philip Morris' approach to regulatory issues [81],[82].

The UK consultancy firm Charles Barker appears to have then been asked to outline the advantages for BAT of embedding RA within UK and European policymaking processes and advised that BAT would need to tread carefully, lobbying through a “front” organisation and enlisting other “big industry names in support” [83]. The chemical and pharmaceutical industries were highlighted as other sectors likely to benefit from (and therefore support calls for) the kind of RA BAT was seeking [83]. In line with this advice, BAT's recruitment efforts appeared to focus solely on companies manufacturing regulated products [55]. By autumn 1996, BAT had commenced discussions with a range of other companies, including SmithKline Beecham, Zeneca, Shell, British Petroleum and Imperial Chemical Industries (ICI), about a campaign to promote RA and IA/CBA as mandatory processes for all proposed EU regulation [57],[58],[84] (for more details about the companies that became involved to varying degrees, see Table 2). Stuart Chalfen (BAT) claimed there was “a real possibility that, with push from around the EU by interested companies, particularly through their trade associations, a basis will be established on which structured risk assessment within cost benefit analysis could be established as the paradigm for European regulation in the future” [57]. He went on to argue that such a development would represent a “remarkable step forward for the future of our companies” [57].

**Table 2:** Other companies involved in the campaign to achieve regulatory reforms in Europe.

Companies (other than BAT) Which Were Members of the EPC Risk Assessment Forum and/or Which Participated in EPC Risk Assessment Forum Events [55],[58],[99],[107],[113],[272]	Business Groups and Companies (Other than BAT) That Were Involved in Fair Regulation Campaign Meetings and/or Email Discussions [205],[214]
• Baxter	*Business & other groups:*
• Bayer	• British Chambers of Commerce
• Bouygues	• Confederation of British Industry (CBI)
• Coca Cola	• Engineering Employers Federation
• Dow	• Federation of Small Businesses
• Du Pont de Nemours	• National Council for Voluntary Organisations
• Edelman	• UK Offshore Operators Association
• Elf Aquitaine	*Companies:*
• GCPF (Crop Protection Association)	• British Telecom (BT)
• HSBC	• British Aerospace
• Imperial Chemicals Company (ICI)	• British Airways
• Johnson & Johnson	• British Nuclear Fuels Limited (BNFL)
• Marks & Spencer	• Carlton
• Mars	• Chantrey Vellacott
• NatWest	• Clifford Chance
• Pirelli	• Cogéma (now part of AREVA NC)
• Shell*	• Covington & Burling
• Siemens	• Diageo
• SmithKline Beecham	• Enron
• Solvay	• ICI
• Tesco	• KPMG
• Zeneca*	• Linklaters & Alliance
	• Lovell White Durrant
	• Mobil
	• NTL (National Transcommunications Limited)
	• Proctor & Gamble
	• Rolls Royce
	• Telewest
	• Unilever
	• United News & Media
	• Zurich financial services

*Companies that were working with BAT on this campaign from an early stage [55].

BAT thereby ignored Charles Barker's warnings that “eventual guidelines might work against rather than help the tobacco industry's interests,” as they could be “hijacked by other lobbies,” such as environmental campaigners, and “might further confirm the hazards of ETS” [83]. The company seemed to be aware that RA might work against its interests [85] but it appears that, by driving the agenda, BAT hoped to ensure that decisions about how much risk is acceptable were based on a methodological approach that would work in its favour [56],[86].

A proposal from Covington & Burling in February 1996 advised that existing (relatively weak) commitments to IA in Europe could be used to promote a far more specific form of BIA, intended to ensure that new legislation could only be implemented if it could be demonstrated to “achieve significant risk reductions at reasonable cost” [87]. In other words, as well as providing a “policy window” through which RA could be promoted [88], the use of IA/CBA as a framework for RA involved promoting a tool in which reductions in risks to human health (and the environment) would be partially assessed on the basis of their *economic* efficiency. Further potential strategic value of IA (as opposed to RA) is set out in promotional material of IMPACT, a group specialising in IAs that was formed by a UK consultancy group called the Public Policy Unit (PPU) and business economists London Economics [89], which BAT kept on its files [89]–[92]. After stating that IA, “offers business and industry a major new opportunity to influence the policy and legislative process” [91], IMPACT claimed that companies could use IA to “work the system” by: (i) opposing or amending policy proposals; (ii) promoting industry's legislative or regulatory proposals; and (iii) challenging government to review existing regulatory or legislative burdens [91]. The document goes on to suggest that IA can also be used to ensure early consultation with business, to direct policymakers to industry's preferred sources of data, and to delay or block legislation [91]. Significantly, IMPACT's material also suggests that businesses might be able to influence the methodologies of official IAs [91], a possibility also highlighted in a report for BAT by Ernst & Young, one of the world's largest consultancy firms [93]. It is unclear from the data whether BAT first suggested to PPU that IA provided a potential means for corporate policy influence or vice versa. In either case, BAT seems to have been persuaded by IMPACT/PPU's pitch because, in addition to enlisting PPU to undertake a BIA on its behalf [94]–[96], BAT subsequently launched a large-scale and multifaceted campaign aimed at shaping policy approaches to IA in the UK and Europe [97].

### Manufacturing a Supportive Policy Network

The analysis of BAT's internal documents indicates that the preliminary aim of BAT's campaign was to make RA and CBA legally binding within EU policymaking by securing changes to the EU Treaty [98]. To this end it was deemed necessary to create “a large reservoir of informed and favourable opinion towards the project across the EU” [99] at both national and European levels. In seeking to achieve this, from 1996 onwards BAT appears to have relied heavily on the European Policy Centre (EPC) [62],[98],[100]–[115], sometimes working with the Weinberg Group [116]–[118], a US based consultancy firm that had also been involved in Philip Morris' US and international ETS campaign [73],[119]–[125]. The EPC (originally Belmont EPC, referred to in some documents as “Belmont”) is a leading Brussels think tank [126], which Stanley Crossick helped establish in 1991 [127] (BAT had been in contact with Crossick since the late 1980s [128]–[133]) and BAT remains a member today [134].

It seems clear that BAT approached the EPC, which then formed the Risk Assessment Forum (herein referred to as the Forum) on behalf of BAT and its allies, and that both BAT and EPC were then involved in recruiting further companies to join the Forum and thus the broader campaign for regulatory reform (see Table 2 for companies that became involved and/or participated in email discussions about the campaign) ([135],[136]; interview with Stanley Crossick on 18th September 2008). Documents summarising the collective intention of the Forum suggest that its members hoped to promote a new approach to assessing risk that would reduce policymakers' ability to use the precautionary principle (see Table 1) as a basis for EU legislation [107],[137]. For example, one document explains that the Forum “should attempt to define the principles which must be applied to the process of governance of risk rather than leave it to the decision-makers” [107].

In recruiting the EPC and expanding the coalition of corporations, BAT's aim appears to have been to deliberately manufacture a supportive “policy network” [15]–[17], thereby increasing both the credibility and the size of the campaign, and obfuscating the tobacco industry's specific interests in risk assessment [83],[138]. The value of EPC assuming a central role in the campaign to promote IA and RA resides in its perceived status as an independent and highly credible policy actor, which meant the campaign for regulatory reform was far more likely to seem convincing to policymakers:

“I would be absolutely astonished and would find it very difficult to believe if there was any information available which tended to indicate that the EPC was advocating on behalf of the tobacco industry—that would be shocking.” (Interview with David Byrne, Commissioner for Health and Consumer Protection 1999–2004, 27th August 2008)

### Securing Changes to the EU Treaty

To ensure that RA and CBA became legal requirements in EU policymaking [56],[62], our analysis demonstrates that the campaign had to move quickly to influence the EU Treaty via the 1996–1997 Inter-governmental Conference (IGC) [100] (IGCs are the lengthy process via which Member States consider changes to the EU Treaty and the 1996–1997 IGC culminated in the Treaty of Amsterdam [39]). In seeking to influence this process, BAT and the Forum appear to have exploited the multiple entry points that characterise EU policymaking [139]. The companies involved (see Table 2) acted directly, through various third party organisations (such as the EPC and PPU), and through business organisations such as UNICE (Union of Industrial and Employers' Confederations of Europe, now known as BusinessEurope) and the CBI (the Confederation of British Industry), as well as the IBEC (Irish Business and Employers Federation), which was another member of UNICE [98],[105],[140]–[145]. BAT's internal documents indicate that the company was a paying member of the CBI [146]–[151] and that BAT staff held key CBI posts, such as sitting on and chairing various CBI committees [151]–[154]. UNICE, which is now known as BusinessEurope, is the collective body of member states' employers' groups (the UK's representative being the CBI and Ireland's representative being the IBEC) [155],[156] and, hence, BAT was linked into UNICE via its membership of the CBI. There is no evidence to suggest BAT was a direct member of the IBEC, but both the IBEC and the CBI were members of UNICE, and the documents indicate that Stuart Chalfen, of BAT, held a post in UNICE circa 1996 [157]–[159]. Efforts focused on Member States predisposed to favour IA/CBA, such as the UK and Germany [62],[87],[99],[140],[144],[145],[160], as well as Ireland and The Netherlands, which consecutively held the EU Presidency during the IGC (see Table 3) [57],[59],[98],[99],[136],[161]. Both the EPC [162] and UNICE [163] made direct submissions to the IGC that called for CBA to be made legally binding. Although it is possible that some of the groups involved in this “policy network,” particularly UNICE, which was already supportive of CBA in 1995 [164], might have attempted to promote IA/CBA even without BAT's involvement, the EPC appears to have played an important coordinating role during the IGC, helping to ensure that lobbying was carefully directed through a range of organisations and member-states.

**Table 3:** Timeline of six-month rotating EU Presidencies during and immediately after the 1996–1997 Inter-governmental Conference.

Event	Dates	EU Presidency
In 1996 an IGC (the formal name for Member State discussions concerning potential changes to the EU Treaty) was launched in Turin, Italy. After more than 18 months of discussions, and four different EU Presidencies, the IGC eventually culminated in the Treaty of Amsterdam, which was officially signed on 2^nd^ October 1997.	Jan–Jun1996	Italy
	Jul–Dec 1996	Ireland
	Jan–Jun 1997	Netherlands
	Jul–Dec 1997	Luxembourg
Once the Treaty change had been secured in the Treaty of Amsterdam, BAT focused on organising (with the EPC and Weinberg Group) a conference, sponsored by the UK EU Presidency, to further promote RA and IA. This event was used to target Austrian officials (as well as others), as Austria was about to take over the EU Presidency.	Jan–Jun 1998	UK
	Jul–Dec 1998	Austria

Changes to the EU Treaty indicate that these large-scale lobbying efforts were effective ([62],[165]; interview with Stanley Crossick on 18th September 2008). In what was described by BAT as an “important victory” for the company [62] (see Box 1), a Protocol, entitled *Protocol on the application of the principles of subsidiarity and proportionality* and tabled by the UK delegation [166], was appended to the Treaty of Amsterdam [39]. This included provisions calling on the European Commission to “consult widely” and minimise the potential “burden” of policy changes on “economic operators” (and others). This appears to have been interpreted by the EPC and BAT to mean that a form of BIA was now mandatory within EU policymaking [62].

**Box 1. Extracts from an Internal BAT Document [62] Outlining BAT's Influence on the Treaty of Amsterdam (Italics Indicate Our Emphasis)**

**The objective**

*To promote the introduction of legislation which forces governments to justify properly and openly the costs and benefits of their proposals.* In particular, to require governments to undertake a transparent and rigorous assessment of the risks that they are seeking to eliminate:

- For a public smoking ban, government must show a convincing case for the claim that ETS is harmful to the health of non-smokers and that a ban would deliver significant health benefits
- For an advertising ban, government must show a convincing case for the claim that bans reduce the incidence of smoking, including youth smoking

**The opportunity**

*In no country in the world are governments required, in practice, to justify their actions through effective cost-benefit analysis, underpinned by rigorous risk assessment. An opportunity to promote such a requirement was identified in the European Union (EU)*.

The Treaty of the EU does not currently contain any general requirement that government authorities carry out a cost-benefit analysis or structured risk assessment before imposing legislation. However, the EU Treaty was re-negotiated in June 1997 at the Intergovernmental Conference.

*British American Tobacco and BAT Industries recognised that a broad coalition of like-minded companies might be able to persuade member states into amending the Treaty, imposing a binding requirement for cost benefit analysis and risk assessment*.

**The strategy**

British American Tobacco and B.A.T Industries assembled a group of companies with a common interest in rigorous cost benefit analysis and risk assessment.

Supported by a public affairs consultant (European Policy Centre) and a technical consultancy (Weinberg Group), this ad hoc group of companies used its contacts and influence to promote the cause of cost benefit analysis and risk assessment.

Throughout late 1996 and 1997, the campaign gained momentum through lobbies of member state governments, companies, trade organisations, the European Commission and others.

*Germany, the UK, Ireland and the Netherlands (who held the EU Presidency) were identified as the key players and lobbying focused on interests in those states.* […]

**The outcome**

*The new Treaty of the EU includes a (legally binding) Protocol* on subsidiarity (the need to push decision making as far down as possible). Chapter 9 states: Without prejudice to its right of initiative, *the Commission should*:

- Except in cases of particular urgency or confidentiality, *consult widely before proposing legislation* and wherever appropriate, publish consultation documents:
- justify the relevance of its proposals with regard to the principle of subsidiarity; whenever necessary, the explanatory memorandum accompanying a proposal will give details in this respect. The financing of Community action in the whole or in part from the Community budget shall require an explanation;
- *take duly into account the need for any burden, whether financial or administrative, falling upon the Community, national governments, local authorities, economic operators and citizens to be minimised and proportionate to the objective to be achieved;*
- submit an annual report to the European Council, the Council and the European Parliament on the application of Article 3b of the Treaty. This annual report shall also be sent to the Committee of the Regions and to the Economic and Social Committee.

*So, the Commission must now take into account the financial and administrative burden (cost), which has to be minimised and proportionate to the objective (benefit).*

**Next steps (in Europe)**

*A framework now exists in the EU which demands cost benefit analysis and risk assessment*.

The new Treaty comes into force in January 1999. Between now and then *it is vital that pressure is kept up to ensure that the EU does not get off the hook and that the new Protocol is translated into a meaningful system of regulation.* There are two areas of focus:

- To promote the wider acceptance and understanding of the need for structured risk assessment as part of a cost benefit analysis regime
- To ensure that the Commission introduces measures which clearly, efficiently and reliably deliver on the Treaty's promise.

Strategy discussions are continuing with the other companies. For example, seminars on risk assessment for legislators, business interests and others are already being planned, with particular advantage being taken of the British presidency in early 1998. Also being developed are plans to input at every opportunity to the Commission's development of cost benefit analysis and risk assessment practices. […]

**Next steps at British American Tobacco**

*British American Tobacco has achieved an important victory in a key trade bloc. A priority should now be to encourage and empower other parts of the world to embark on similar exercises.*

Science and Regulation propose developing a best practice tool which explains the benefits to British American Tobacco markets of achieving a government requirement for rigorous cost benefit analysis and structured risk assessment. It will also detail the goals, approach, strategy and outcomes of the European campaign, as an example of what can be achieved.

*Our company has an enormous amount to gain* from soundly based regulatory environments. The achievements that have been realised in the EU provide an important stepping stone.” [62]

### Embedding the Treaty Changes within Policymaking Processes

#### The central role of the UK government

Our analysis suggests that continued political activity around RA and IA was deemed essential by BAT and the EPC to embedding the Protocol's principles in the EU policymaking process as the companies involved were uncertain how officials would interpret the Protocol [167]. Documents show that the Treaty change was therefore used to enhance lobbying activities and encourage other companies to join the campaign to promote RA and IA [168].

Since the UK submitted the Protocol and was perceived as the Member State most committed to IA, the UK's assumption of the EU Presidency for six months from January 1998 was regarded as an important “window of opportunity” [165]. As part of its strategy to guide officials' approach to IA and RA, BAT helped organise a conference (in April 1998), assisted by the EPC and Weinberg Group and supported by other companies [84],[103],[112],[116],[135],[168]–[172]. Seemingly drawing on the American campaign to redefine RA, BAT recruited Steve Milloy as a keynote speaker [173],[174]. Milloy was Executive Director of The Advancement of Sound Science Coalition (TASSC), which was linked to Philip Morris' campaign to discredit scientific findings that were not in its interests [73], as part of efforts to denigrate the Environmental Protection Agency's (EPA's) approach to RA [66], and which BAT had also begun to work with in 1997 [175]–[177]. Despite the fact that BAT played a substantial role in the conference [173],[178]–[181], its involvement appears to have remained well hidden [116],[117],[182]–[184], particularly as the UK's EU Presidency appears to have been persuaded to officially sponsor the event (by officially sanctioning it, rather than providing funding, which BAT had already agreed to pay [185]) [135],[186],[187].

In supporting the conference, the UK government provided crucial political legitimacy to BAT's campaign for regulatory reform. The role of David Clark MP, who as Chancellor for the Duchy of Lancaster between May 1997 and late 1998, was the minister responsible for regulatory reform in the UK, appears to have been central. Clark was already well-known to BAT by the time he was appointed to this ministerial position [188]–[196]. Before 1992, Clark had several meetings with BAT representatives [188],[190],[191] and, following a June 1992 BAT Chairman's Policy Committee, the minutes of which note that Clark “would be appointed as a political advisor to the Company” [197], Clark attended several high-level BAT meetings [185],[189],[192]–[194]. In 1995, a BAT member of staff reported in an internal memo that Clark had offered to lobby other MPs on issues relating to economic impact studies on BAT's behalf [198], suggesting that by the time Clark entered the Cabinet in May 1997 he was not only aware of BAT's interest in IA but appeared to be supportive of the company. It is unclear whether or not Clark was specifically aware of BAT's involvement in the April 1998 UK Conference although shortly prior to this conference, Stuart Chalfen (of BAT) had written to Clark requesting “a little time together” [157] at another conference on regulatory issues organised by Clark [199], at which Chalfen had been invited to speak [157],. Whether or not Clark knew of BAT's involvement in the April conference, he agreed to support the conference [168],[200] and was also involved in other efforts to promote regulatory reform during the EU Presidency [201]. Bruce Ballantine (of EPC [202]) was involved with one or more key advisory groups organised by Clark, one of which was described as a task force on “open government,” designed to feed into a white paper on regulatory reform [135], and another which was referred to as the “Better Government Task Force,” into which Ballantine offered to feed comments from the Forum [203] (it is unclear whether or not these groups were one and the same).

#### The Fair Regulation Campaign

Additionally, the documents show that BAT and other companies came together to form the UK-based Fair Regulation Campaign (FRC) in January 1999, with the stated intentions of: (i) ensuring that BIA for all policy initiatives proposed by the UK government, the EU, or associated regulatory bodies was legally required (and monitored); and (ii) maximising consultation with business in relation to BIA (including on methodology and data) [204],[205]. At least two internal BAT documents provide evidence of BAT providing financial support to the FRC [206],[207]. The Director of the FRC was Charles Miller of PPU [204],[208]–[211], a consultancy company with which BAT had extensive links [97],[160],[212],[213]. The creation of another, ostensibly unrelated, constituency lobbying for regulatory reform is likely to have further obfuscated BAT's specific interests and enhanced the credibility of the campaign. The FRC quickly won the support of Erkki Liikanen (then Commissioner for Enterprise and Information) [209],[214]–[216] and a number of other Commissioners who were involved in the “Better Regulation” impetus in Europe (interview with Charles Miller on 27th August 2008).

#### From BIA to consultation with stakeholders

Commissioner Liikanen went on to oversee a pilot study of the BIA process in Europe [217] for which EPC was commissioned to produce a contributory paper. Published in September 2001 [218] the EPC paper argued that the system for undertaking IAs in the European Commission was inadequate and that a lack of stakeholder consultation was a key concern, a theme taken up in the subsequent BIA pilot study report that recommended “Key minimum standards for consultation should be implemented for all consultation activities that include dialogues with stakeholders and interested parties” [219]. The EPC paper acknowledges that Christopher Proctor (who was by then BAT's head of science and regulation) had acted as Chair of the EPC's Risk Forum Steering Group and that the Forum had been significantly involved in producing the report [218]. In what appears to be a direct response to the recommendation for consultation standards (as well as a broader reaction to a 1999 scandal in which 20 Commissioners resigned over allegations of nepotism and corruption [220]) the European Commission subsequently published minimum standards of consultation [221], which took effect on 1st January 2003.

### Subsequent Industry Use of IA within Europe

The tobacco industry has already used IA commitments and the requirement for stakeholder consultation to actively challenge EU tobacco control legislation in a number of ways. In the case of the EU's Tobacco Products Directive, the industry argued that both the European Commission and the UK government failed to conduct adequate IAs [222]–[224]. In the UK, the Tobacco Manufacturers' Association, of which BAT is a leading member, responded to the official draft regulatory impact assessment (RIA) by providing alternative (higher) compliance costs [225] and claiming that the government failed to consult properly [223],[224]. BAT itself took a similar approach in a report it produced which overplayed the potential job losses (and related economic costs) associated with this legislation [226]. This approach echoed similar challenges to the UK's implementation of EU legislation restricting tobacco advertising [142],[227]–[229], which resulted in the then Minister for Public Health being called before the European Legislation Select Committee [142],[228],[230] and a full RIA being subsequently published, which BAT and its allies then challenged [231]–[240]. More recently, tobacco companies have been using the requirement for stakeholder consultation laid out in the European Commission's minimum standards on consultation [221] and IA guidelines [241],[242] to lobby against Article 5.3 of the World Health Organisation's Framework Convention on Tobacco Control [243]–[246] (the Framework Convention is a global health treaty, which the EU has signed and ratified, and Article 5.3 specifically seeks to protect public health policies from tobacco industry interference [247]). Imperial Tobacco has recently employed very similar arguments to lobby against Article 5.3 in the UK [248].

Whilst these attempts so far appear only to have delayed EU tobacco control legislation, not to have prevented or significantly weakened it, several of our interviewees (both those who were involved in the lobbying campaign described in this paper and those working for public health and environmental NGOs) stated that similar strategies were more likely to succeed now that IA has been further embedded within EU systems. Importantly, these claims are supported by the fact the chemical industry, which was also involved in the campaign described in this paper, has more recently used the EU's IA system to successfully delay and weaken legislation intended to protect the public's health and the environment (see Box 2). The most recent IA-based challenge by a tobacco company (of which we are aware) was to write to the Chairman of the European Commission's internal Impact Assessment Board in July 2008 to complain that the IA on smoke-free environments, which has yet to be published, did not fully comply with the European Commission's IA guidelines and that the process of stakeholder consultation was inadequate [245]. This issue is ongoing and it remains to be seen whether tobacco companies' IA-based lobbying will influence developments.

**Box 2. Chemical Industry Use of IA to Delay and Weaken EU Legislation Intended to Protect Public Health**

To date, chemical companies appear to have been more successful than tobacco companies at employing IA in the EU, having employed IA to delay and weaken EU regulation on the Registration, Evaluation, Authorisation and Restriction of Chemical Substances (REACH) [273]. Given mounting evidence of the strong link between chemical products and degenerative diseases of the central nervous system and cancers [274]–[278], REACH, which is underpinned by the precautionary principle, is potentially one of the most important pieces of legislation ever passed by the EU. The intention was that it would reverse the burden of proof, making companies (rather than regulators) responsible for providing data to support safety claims (including for chemicals already in use), and require mandatory substitution for some of the most hazardous chemicals on the market. However, the chemical industry was able to dilute key aspects of the proposed regulation, including the requirement for mandatory substitution of hazardous chemicals [279], and there is evidence that the European chemical industry's use of IAs played a crucial role in this process.

After the European Commission launched an internet consultation on REACH in May 2003, the industry contracted a number of consultants to produce IAs as a way of challenging some of the proposals [280],[281]. These IAs greatly exaggerated the costs associated of the proposed legislation and, although this was recognised [282], they have nevertheless been credited with shaping policymakers' perceptions of the prospective costs and benefits of REACH [283]–[285]. More significantly, the industry's wide use of consultancy firms to undertake IAs on its behalf [283] meant the independence of many of the firms with experience in conducting large-scale IAs in Europe was compromised, restricting the choices available to EU institutions, which did not at that time have sufficient resources to conduct an IA on REACH internally. Hence, when DG Research commissioned Arthur D. Little (ADL) (a company with a history of working for the tobacco industry [10],[286]–[290]) to produce an official IA [291], the company was already working on an IA for the German chemical industry [292]. ADL's report for the European Commission used the same parameters and methods of calculation as its report for the German industry and estimated costs to the industry to be somewhere between 300 and 600 times greater than the most conservative estimates of the European Commission [291],[293]. Despite serious questions over the reliability of this IA [282],[293] intense corporate lobbying [294] subsequently led to a Memorandum of Understanding between the industry, DG Enterprise, and DG Environment [295], which effectively resulted in three industry sponsored IAs [296]–[298] being incorporated within the European Commission's IA framework [299],[300]. To ensure confidence in the process, a number of NGOs were invited to join a working group designed to monitor these IAs but, in July 2004, two withdrew their support, claiming that the study methods lacked transparency, were inconsistent and imbalanced, and placed undue focus on business risks [294].

In the meantime, lobbying efforts relating to regulatory reforms are ongoing [21],. The EPC, of which BAT and other tobacco companies remain members [134], are increasingly, and apparently successfully [18], depicting the precautionary principle as inconsistent with scientific approaches to policymaking [21],[250].

## Discussion

### Key Findings

Our findings indicate that, having monitored a similar campaign by Philip Morris in the US [63],[66], senior managers at BAT initiated a large-scale, multifaceted lobbying campaign in Europe that quickly helped secure an important and legally binding amendment to the EU Treaty, providing a constitutional platform for BIA to be embedded within EU policymaking procedures—changes that a BAT document describes as an “important victory” for the company [62]. Subsequent lobbying efforts appear to have helped promote and embed a system of IA in the EU that is business orientated, subsuming RA within it and encouraging policymakers to consult businesses. We conclude that, in doing so, BAT and other large companies operating in Europe have fundamentally altered EU policymaking by ensuring that all decisions are passed through an economic framework that provides business with a range of advantages [251].

To achieve the reforms it desired, BAT appears to have specifically recruited other industries (sometimes directly and sometimes through the EPC) that, by virtue of the hazardous nature of their products, shared an interest in challenging the precautionary principle as a basis for regulation. In other words, BAT deliberately helped manufacture a “policy network” to enhance the extent to which the regulatory reforms it wanted to promote would appear credible (and not partisan), with the intention of increasing the likelihood that these reforms would be successfully implemented. In addition, by operating through a major think tank (the EPC) and a purposefully established campaign group (the FRC), BAT seems to have been able to distance itself from the push for IA and RA. The fact that our interviews with European Commission staff revealed no awareness of tobacco industry involvement in campaigns for IA (or of the extent to which business interests may be privileged through IA) corroborates claims, made elsewhere [252],[253], that policymakers may be unaware of how changes to policy are taking effect or who is behind them, an issue which may be particularly pertinent in the EU, which is a complex political system with multiple points of access [139].

As BAT and other companies in the EPC Forum planned [161],[254], there has been a significant shift in how risk is conceptualised within Europe, which has helped effect a move away from the precautionary principle towards IA [18] and towards the individualisation of risk [255]. These changes are particularly concerning given that BAT's intention was to prevent legislation intended to protect the public's health. As this paper illustrates, these reforms have already been employed to challenge legislation intended to protect public health. If regulatory reforms continue to move in this direction, as many, including our interviewees, have predicted [18],[256], there is a real danger that the burden of proof will shift further towards those trying to manage risks and away from those who profit from the production and sale of risky goods. Our interview data indicate that to date corporate actors have not yet challenged EU legislation in the courts on the primary basis that the IA process was insufficient. Nor have business-sponsored IAs yet been widely used to challenge existing (rather than forthcoming) legislation. However, interviews with business lobbyists and analysis of BAT documents [91],[257],[258] suggest that both potentialities are under consideration.

Overall, the evidence presented here supports key concerns previously raised about IA. It shows that, in practice, IA is far from the transparent and rational process described by advocates and can instead serve to enhance corporate influence over health policy, as the material from IMPACT cited in this paper explicitly describes [91]. Indeed, its incorporation into EU decision-making has been promoted by major European businesses for this very reason [256]. The findings therefore help explain why independent assessments of the European Commission's IAs have consistently found that economic impacts have received more attention than environmental [43],[44] or social (and particularly health) impacts [43]–[47],[259].

### Implications for Public Health and Policymakers

Given that contemporary public health problems are increasingly being linked to the activities of large corporations, our findings have important consequences for public health. First, they confirm that corporate influence over health policy can extend well beyond the immediate remit of a particular sector's product; public health groups and policymakers therefore need to give more attention to corporate efforts to shape decision-making processes as well as specific outcomes. In the current European context, and in light of the ambitions of some of the companies involved, further analysis is required to explore how large corporations are attempting to reframe debates about risk and the costs of legislation.

Second, the findings suggest the public health community's positive acceptance of IA (focusing on HIA) [260],[261] ought to be reconsidered [251]. This is particularly important in the context of the private sector's well-documented ability to use its resource advantage to shape both scientific knowledge and public understandings of science by consistently challenging the method of robust scientific studies and by misrepresenting the findings of industry-funded science [262]–[266]. In light of these factors, it is important to consider whether tools such as IA and CBA are inevitably flawed from the perspective of those interested in maximising human (or environmental) well-being, as some have claimed [26],[27], or whether it is possible to employ IA tools in ways that do not systematically advantage corporate interests. Further, comparative research is required to investigate this issue by exploring whether IA processes are prioritising business interests (and underplaying health impacts) to the same extent elsewhere. In the meantime, a review of the current system of IA operating in the EU is urgently required. One possible starting point would be for the European Commission to reopen the relatively short consultation period for its most recent guidelines on IA, which ran between June and July 2008 [267], and to ensure that this is now more widely publicised. Finally, given that the EU Treaty also requires the EU to “ensure a high level of human health protection” in all its activities, perhaps it is time for the public health community to officially challenge the European Commission's failure to undertake HIAs of its policies [45].

Combined, these issues underline the need for public health groups in Europe to have better information from, and representation in, Brussels given the important influence that EU developments will have on national public health policy. Moreover, in light of the apparent success of BAT's attempts to influence EU policy by establishing a pan-industry “policy network,” our results suggest that it may be more effective, and efficient, for different interest public health groups to collaborate more closely in their efforts to influence policy.

## Supporting Information

Alternative Language Abstract S1 French translation of the abstract by Florence Berteletti Kemp.
(0.11 MB DOC)

Alternative Language Abstract S2 German translation of the abstract by HW.
(0.03 MB DOC)

Alternative Language Abstract S3 Italian translation of the abstract by Massimo Giornetti.
(0.03 MB DOC)

Alternative Language Abstract S4 Portuguese translation of the abstract by Sandra Tavares Moreira.
(0.07 MB DOC)

Alternative Language Abstract S5 Spanish translation of the abstract by Sandra Tavares Moreira.
(0.08 MB DOC)

## Abbreviations

BAT: British American Tobacco
BIA: business impact assessment
CBA: cost benefit analysis
CBI: Confederation of British Industry
DG: Directorate General
EPC: European Policy Centre
ETS: environmental tobacco smoke
EU: European Union
FRC: Fair Regulation Campaign
HIA: health impact assessment
IA: impact assessment
IBEC: Irish Business and Employers Federation
ICI: Imperial Chemical Industries
PPU: Public Policy Unit
RA: risk assessment
REACH: Registration, Evaluation, Authorisation and Restriction of Chemical Substances
RIA: regulatory impact assessment
TMA: Tobacco Manufacturers' Association
UNICE: the Union of Industrial and Employers' Confederations of Europe (now known as BusinessEurope)
