# Supplementary material for: “Working the System”—British American Tobacco's Influence on the European Union Treaty and Its Implications for Policy: An Analysis of Internal Tobacco Industry Documents
Source: PLoS Med. 2010 Jan 12;7(1):e1000202. doi: 10.1371/journal.pmed.1000202 (PMC2797088; doi:10.1371/journal.pmed.1000202)
Supplement: Alternative Language Abstract S1 — French translation of the abstract by Florence Berteletti Kemp. (0.11 MB DOC) [file pmed.1000202.s001.doc]

**« Au cœur du système » - Quelle est l’influence exercée par British American Tobacco sur le traité de l’Union européenne et quelles sont les conséquences d’une telle influence sur les politiques communautaires ? Une analyse basée sur la documentation interne de l’industrie du tabac.**

**Le contexte :** La réalisation d’une analyse d’impact (AI) pour l’ensemble des principales politiques de l’Union européenne (UE) est désormais obligatoire. Le type d’AI actuellement utilisé fait l’objet de critiques, car il favoriserait les intérêts des grandes entreprises, en accordant trop d’importance aux impacts économiques et trop peu à une évaluation satisfaisante des répercussions sanitaires. Notre étude cherche à comprendre comment, pourquoi et de quelle manière, les entreprises (notamment les grandes entreprises comme l’industrie du tabac) ont réussi à influencer l’approche adoptée par l’UE dans le domaine des analyses d’impact.

**Méthodes et constatations :** Afin de déterminer si l’industrie a joué un rôle dans la promotion du recours systématique aux AI au sein de l’UE, nous nous sommes livrés à une analyse de la documentation interne de British American Tobacco (BAT), qui a été rendue publique suite à une série de cas de litiges intervenus aux États-Unis. Notre analyse s’appuie également sur la littérature et les interviews concernant les acteurs impliqués dans ce domaine. Notre analyse permet de démontrer qu’à partir de 1995, BAT a coopéré de manière active, ainsi que d’autres grandes entreprises, à la promotion d’un certain type d’AI formulé en termes économiques (c’est-à-dire sous la forme de l’analyse coûts-bénéfices, ou ACB), qui favorise les grandes entreprises.

Les dirigeants de BAT étaient d’avis que ce type d’AI permettrait de faire progresser les intérêts des entreprises européennes, et de mettre en place des procédures de définition des politiques qui : (i) définiraient un cadre économique en vue de l’évaluation de l’ensemble des décisions politiques, accordant la priorité, de manière implicite, aux coûts impliqués pour les entreprises ; (ii) garantiraient la participation en amont des grandes entreprises dans les débats politiques ; (iii) accorderaient aux grandes entreprises un avantage à long-terme par rapport aux autres acteurs, et rendraient les responsables politiques toujours plus dépendants des informations fournies par ces grandes entreprises ; (iv) mettraient à disposition des entreprises des moyens de persuasion permettant de contester les réglementations en vigueur ou envisagées. Les données consultées mettent en évidence que la campagne de lobbying qui s’en est suivie, largement menée par BAT, a en effet contribué à ce que des modifications contraignantes soient inscrites au traité sur l’Union européenne, par le biais du traité d’Amsterdam, qui obligent les responsables politiques de l’UE à minimiser les obligations juridiques pesant sur les entreprises. Une fois obtenue ce que BAT a décrit comme une « victoire importante », leurs efforts se sont concentrés sur la mise en œuvre de ces dispositions contraignantes en appliquant systématiquement aux procédures de décisions communautaires le recours au type d’AI voulu, c’est-à-dire celui favorable aux entreprises (l’ACB). L’industrie du tabac comme l’industrie chimique ont, depuis, recours à ce type d’AI, dans le but de mettre en cause les éléments clés des politiques communautaires de protection de la santé publique.

**Conclusion :** Ces constatations nous permettent de penser que BAT, et ses alliés issus du secteur économique, ont réussi à modifier fondamentalement les méthodes de formulation et d’élaboration des politiques de l’Union européenne, en rendant obligatoire le recours aux types d’AI favorables à la logique entrepreneuriale. Le fait que la totalité des décisions politiques importantes adoptées au sein de l’UE doivent, désormais, être évaluées au moyen d’une telle méthode, fondée sur la technique de l’ACB, accorde aux grandes entreprises un avantage unique. Cet état de fait vient accroître la probabilité que les politiques adoptées par l’UE privilégient les intérêts des entreprises les plus puissantes (y compris celles qui produisent des produits nocifs pour la santé) et non ceux des citoyens. La communauté de la santé publique, dont le travail est guidé par les résultats des AI en matière de santé, a, en effet, accueilli de manière favorable le nouvel intérêt porté aux AI pour l’élaboration des politiques. Il semble donc aujourd’hui urgent de réfléchir à la façon dont chaque type d’AI existant peut être utilisé soit pour affaiblir, soit pour soutenir, les politiques de santé publique.
