# Supplementary material for: “Working the System”—British American Tobacco's Influence on the European Union Treaty and Its Implications for Policy: An Analysis of Internal Tobacco Industry Documents
Source: PLoS Med. 2010 Jan 12;7(1):e1000202. doi: 10.1371/journal.pmed.1000202 (PMC2797088; doi:10.1371/journal.pmed.1000202)
Supplement: Alternative Language Abstract S2 — German translation of the abstract by HW. (0.03 MB DOC) [file pmed.1000202.s002.doc]

**Der Einfluss von British American Tobacco auf den Vertrag über die Europäische Union und dessen Folgen auf politische Entscheidungsfindung: eine Analyse interner Tabakindustriedokumente**

**Hintergrund**: Im Vertrag über die Europäische Union ist die Durchführung von Folgenabschätzungen aller europäischen Richtlinien gesetzlich verankert. Die vorgesehene und praktizierte Form der Folgenabschätzung wird jedoch dafür kritisiert, dass sie einerseits die ökonomischen Folgen unverhältnismäßig stark betont, während die gesundheitlichen Folgen nicht ausreichend berücksichtigt werden. Unsere Studie stellt heraus, wie und aus welchen Gründen British American Tobacco (BAT) die Durchführung von Kosten-Nutzen-Analysen in der Europäischen Union vorangetrieben hat. Unsere Forschungen zeigen auf, dass große Unternehmen unter Leitung von British American Tobacco (BAT) eine wesentliche Gruppe waren, die die Einführung von Folgenabschätzungen in Europa voran getrieben haben, und dass dies ein Hauptgund für den derzeit praktizierten Ansatz in der Europäischen Union ist.

**Methodik und Ergebnisse**: Als Teil eines Forschungsprojektes zur Einflussnahme der Tabakindustrie auf die europäische Politik analysierten wir interne, durch amerikanische Gerichtsverfahren zugänglich gemachte Industriedokumente des Tabakkonzerns British American Tobacco (BAT). Ergänzt wurden diese durch eine Literaturanalyse zu Kosten-Nutzen-Analyse und Folgenabschätzung und durch Interviews mit relevanten Akteuren. Unsere Analyse zeigt, dass BAT mit anderen betrieblichen Akteuren kooperierte, um europäische gesetzliche Reformen im Interesse von großen Konzernen erfolgreich voran zu treiben. Die Bemühungen konzentrierten sich auf die Förderung von betriebswirtschaftlich orientieren Formen von Folgenabschätzung (eine Version der Kosten-Nutzen-Analysen). BAT-Bereichsleiter nahmen an, dass die Verankerung einer solchen Form der Folgenabschätzung Grundregeln für politische Entscheidungsfindung festlege und dadurch die europäischen Interessen der Fima maßgeblich unterstütze. Ziel der Einführung dieser Regeln war: (i) einen ökonomischen Rahmen zu bieten zur Evaluation aller politischen Entscheidungen mit einem implizieten Fokus auf betriebswirtschaftlichen Kosten; (ii) die rechtzeitige Einbindung von Firmen in politische Entscheidungsprozesse sicher zu stellen; (iii) dem betrieblichen Sektor einen langfristigen Vorteil gegenüber anderen politischen Akteuren zu sichern, indem politische Entscheidungsträger stärker von betrieblichen Informationen abhängig gemacht würden; (iv) den Unternehmen überzeugende Mittel zur Verfügung zu stellen, die ihnen ermöglichen, potentielle und vorhandene Gesetzgebung anzufechten.

Die Dokumente enthüllen, dass die folgende, vorrangig durch BAT voran getriebene Lobby-Kampagne half, Veränderungen im Vertrag von Amsterdam zu verankern, die die politischen Entscheidungsträger zwingen, die Last gesetzlicher Entscheidungen für Unternehmen zu minimieren. Nachdem dies erreicht war, was BAT als „wichtigen Sieg“ bezeichnete, konzentrierten sich weitere Bemühungen darauf, die Änderungen des Vertrags in Prozessen der europäischen politischen Entscheidungsfindung umzusetzen und vorrangig die wirtschaftlich ausgerichteten Formen der Folgenabschätzung im Sinne von Kosten-Nutzen-Analysen einzuführen. Sowohl die Tabak- als auch die chemische Industrie haben seitdem Folgenabschätzung angewandt, um Schlüsselaspekte europäischer gesundheitspolitischer Richtlinien zu untergraben.

**Schlussfolgerungen**: Unsere Ergebnisse legen den Schluss nahe, dass BAT und seine unternehmerischen Partner eine grundlegende Veränderung europäischer politischer Entscheidungsprozesse ausgelöst haben. Als Folge der oben beschriebenen Kampagne ist eine unternehmensnahe Form der Folgenabschätzung (die der betriebswirtschaftlichen Kosten-Nutzen-Analyse ähnlich ist) etabliert und gilt als verbindliches Bezugssystem für alle wichtigen politischen Entscheidungen in der Europäischen Union. Diese Situation verschafft großen Konzernen einen einzigartigen Vorteil. Zudem erhöht sie die Wahrscheinlichkeit, dass europäische Richtlinien entwickelt werden, durch die die Interessen großer Unternehmen begünstigt werden. Dies gewinnt an besonderer Relevanz, wenn Produkte hergestellt werden, die die Gesundheit gefährden oder schädigen und das gesellschaftliche Interesse negieren. In Anbetracht der Tatsache, dass sich gesundheitspolitische Interessenvertreter auf gesundheitliche Folgenabschätzung konzentriert und den verstärkten politischen Fokus auf Folgenabschätzung begrüßt haben, ist es dringend nötig, dass das Augenmerk verstärkt darauf gerichtet wird, wie Folgenabschätzung eingesetzt wird, um effektive gesundheitspolitische Zielsetzungen zu untergraben oder zu unterstützen.
