# Supplementary material for: “Working the System”—British American Tobacco's Influence on the European Union Treaty and Its Implications for Policy: An Analysis of Internal Tobacco Industry Documents
Source: PLoS Med. 2010 Jan 12;7(1):e1000202. doi: 10.1371/journal.pmed.1000202 (PMC2797088; doi:10.1371/journal.pmed.1000202)
Supplement: Alternative Language Abstract S3 — Italian translation of the abstract by Massimo Giornetti. (0.03 MB DOC) [file pmed.1000202.s003.doc]

**‘Come manipolare il sistema’ - L'influenza della British American Tobacco sul trattato dell'Unione europea e le relative implicazioni sul piano politico: un'analisi di documenti interni dell'industria del tabacco**

**Quadro generale.** La valutazione d'impatto di tutte le principali politiche dell'Unione europea (UE) è ora obbligatoria. Il tipo di valutazione d'impatto impiegata è stata oggetto di critiche in quanto favorisce gli interessi delle imprese ponendo un accento sproporzionato sull'impatto economico senza tener adeguatamente conto dell'impatto sulla salute. Il nostro studio cerca di valutare in che modo, perché e con quali mezzi le imprese, e in particolare l'industria del tabacco, hanno influenzato l'approccio dell'UE alla valutazione d'impatto.

**Metodologia e risultanze.** Per accertare se l'industria ha svolto un ruolo nel promuovere questo sistema di valutazione d'impatto in seno all'UE, abbiamo analizzato documenti interni della British American Tobacco (BAT), divulgati a seguito di una serie di cause intentate negli Stati Uniti, esaminando nel contempo la letteratura in materia e interviste con soggetti interessati. La nostra analisi dimostra che a partire dal 1995 la BAT ha attivamente collaborato con altri soggetti aziendali per promuovere una forma di valutazione d'impatto di stampo imprenditoriale (una versione dell'analisi costi-benefici) tale da favorire le grandi società. I dirigenti della BAT ritenevano che l'impiego di questo tipo di valutazione d'impatto avrebbe promosso gli interessi europei dell'azienda grazie all'introduzione di norme fondamentali in materia di processo decisionale che: i) avrebbero creato un quadro economico per la valutazione di tutte le decisioni politiche, con un'implicita importanza prioritaria per i costi per le imprese; (ii) avrebbero garantito la partecipazione delle imprese alle discussioni politiche fin dalle prime fasi; (iii) avrebbero offerto al settore imprenditoriale un vantaggio a lungo termine rispetto ad altri soggetti grazie alla crescente dipendenza dei responsabili politici dalle informazioni da esso fornite; (iv) avrebbero dato alle imprese uno strumento persuasivo per contestare normative in esame e vigenti. I dati raccolti indicano che una successiva campagna di pressione, promossa principalmente dalla BAT, ha contribuito all'inserimento di modifiche vincolanti nel trattato UE, attraverso il trattato di Amsterdam, che imponevano ai responsabili politici di limitare al massimo gli oneri legislativi per le imprese. Dopo aver riportato un successo descritto dalla BAT come "un'importante vittoria", gli sforzi si sono successivamente concentrati sull'obiettivo di tradurre tali modifiche al trattato nell'applicazione di una forma di valutazione d'impatto di stampo imprenditoriale (l'analisi costi-benefici) nel processo decisionale dell'UE. Da allora, sia l'industria del tabacco che il settore chimico hanno utilizzato la valutazione d'impatto per cercare di compromettere aspetti fondamentali delle politiche europee relative alla tutela della salute.

**Conclusioni.** Le nostre constatazioni indicano che la BAT e le imprese alleate hanno sostanzialmente modificato il processo di formazione delle politiche, rendendo obbligatoria una valutazione d'impatto di stampo imprenditoriale. Il fatto che tutte le decisioni politiche importanti dell'UE debbano essere valutate attraverso questo quadro che ricalca l'analisi costi-benefici offre alle grandi imprese un vantaggio unico nel suo genere. Ciò accresce la probabilità che l'UE adotti politiche che favoriscono gli interessi delle grandi società, compresi i fabbricanti di prodotti nocivi per la salute, anziché gli interessi dei suoi cittadini. Dato che la comunità dei soggetti che si occupano di sanità pubblica, interessati a una valutazione d'impatto sanitario, ha ampiamente apprezzato il crescente interesse politico nei confronti della valutazione d'impatto, occorre esaminare con urgenza i modi in cui la valutazione dell'impatto può essere utilizzata per compromettere, oltre che sostenere, politiche efficaci in materia di tutela della salute.
