# Supplementary material for: “Working the System”—British American Tobacco's Influence on the European Union Treaty and Its Implications for Policy: An Analysis of Internal Tobacco Industry Documents
Source: PLoS Med. 2010 Jan 12;7(1):e1000202. doi: 10.1371/journal.pmed.1000202 (PMC2797088; doi:10.1371/journal.pmed.1000202)
Supplement: Alternative Language Abstract S4 — Portuguese translation of the abstract by Sandra Tavares Moreira. (0.07 MB DOC) [file pmed.1000202.s004.doc]

**“O Coração do sistema” – A influência de British American Tobacco no Tratado da União Europeia: análise de documentos internos da indústria do tabaco**

**Antecedentes:** A avaliação de impacto (AI) do conjunto das principais políticas da União Europeia tornou-se obrigatória. O modelo de AI utilizado tem sido criticado por favorecer os interesses das empresas, pois dá uma ênfase excessiva a impactos económicos em detrimento da avaliação adequada de impactos sanitários. O nosso estudo pretendeu avaliar de que maneira, por que motivo e em que medida empresas, e em particular a indústria do tabaco, exerceram influência na abordagem da UE em matéria de AI.

**Métodos e resultados:** A fim de determinarmos se a indústria desempenhou um papel na promoção deste sistema de AI na UE, analisámos documentos internos de British American Tobacco (BAT), divulgados após uma série de acções judiciais nos EUA. Além disso, procedemos à uma análise de documentação conexa e entrevistas com agentes relevantes. A nossa análise mostra que, desde 1995 em diante, BAT tem colaborado activamente com outras empresas a fim de promover um modelo de AI que privilegia aspectos económicos que beneficiam as grandes empresas. BAT parece ter sido favorável a este modelo de AI já que permitia promover os seus interesses europeus mediante o estabelecimento de normas básicas para a elaboração de políticas que: (i) ofereceriam um quadro económico para a avaliação de todas as decisões políticas, dando prioridade implícita aos custos para as empresas; (ii) assegurariam a participação das empresas, tão cedo quanto possível, nos debates políticos; (iii) confeririam ao sector empresarial uma vantagem duradoura em relação à outros actores ao tornar os decisores políticos cada vez mais dependentes da informação fornecida pelo mesmo; (iv) dotariam as empresas de recursos persuasivos para contestarem a legislação potencial e existente. Os dados revelam que uma subsequente campanha de lóbi, em grande parte impulsionada por BAT, contribuiu para obter alterações vinculativas ao Tratado da UE mediante o Tratado de Amesterdão que exigiu que os decisores políticos da UE minimizassem as sobrecargas legislativas sobre as empresas. Subsequentemente, os esforços concentraram-se em garantir que as alterações aos Tratados se transformassem na aplicação de uma forma de AI orientada para interesses comerciais (análise custo-benefício – ACB) no processo de elaboração de politicas da UE. Desde então, tanto a indústria do tabaco como a indústria química recorreram a essa forma de AI com a clara intenção de enfraquecer aspectos-chave de politicas europeias elaboradas para proteger a saúde publica.

**Conclusões:** As nossas conclusões sugerem que BAT e os seus aliados empresariais alteraram profundamente a forma como são elaboradas todas as politicas da UE ao tornar obrigatório um modelo de AI que privilegia aspectos económicos. O facto de todas as decisões politicas da UE terem agora de ser avaliadas mediante este quadro, que se baseia no método ACB, confere as grandes empresas uma vantagem única. Isto aumenta a probabilidade da UE elaborar politicas favoráveis aos interesses das grandes empresas, incluídas as que fabricam produtos nocivos para a saúde, em detrimento dos interesses dos seus cidadãos. Dado que a comunidade de saúde pública, concentrando-se na AI no âmbito da saúde, acolheu com grande satisfação o interesse político crescente por a AI, isto sugere que é necessário estudar com urgência como se pode recorrer a AI para enfraquecer, assim como apoiar, politicas de saúde publica eficazes.
