# Supplementary material for: “Working the System”—British American Tobacco's Influence on the European Union Treaty and Its Implications for Policy: An Analysis of Internal Tobacco Industry Documents
Source: PLoS Med. 2010 Jan 12;7(1):e1000202. doi: 10.1371/journal.pmed.1000202 (PMC2797088; doi:10.1371/journal.pmed.1000202)
Supplement: Alternative Language Abstract S5 — Spanish translation of the abstract by Sandra Tavares Moreira. (0.08 MB DOC) [file pmed.1000202.s005.doc]

**“El corazón del sistema” – La influencia de British American Tobacco en el Tratado de la Unión Europea: un análisis de documentos internos de la industria del tabaco**

**Antecedentes:** La evaluación de impacto (EI) del conjunto de las principales políticas de la Unión Europea (UE) se hizo obligatoria. El modelo de EI utilizado se ha criticado por favorecer los intereses de las empresas, poniendo mayor énfasis en impactos económicos, en detrimento de la evaluación adecuada de impactos sanitarios. Nuestro estudio intentó evaluar cómo, porqué y en qué medida las empresas, y en particular la industria del tabaco, influyeron en el planteamiento de la UE en materia de EI.

**Métodos y resultados:** Para determinar si la industria desempeñó un papel en la promoción de dicho sistema de EI en la UE, analizamos documentos internos de British American Tobacco (BAT), divulgados tras una serie de pleitos que ocurrieron en los EE.UU.. Esto se ha complementado con un análisis de publicaciones conexas y entrevistas con informantes claves. Nuestro análisis demuestra que del 1995 en adelante BAT colaboró activamente con otras empresas a fin de promover la creación de un tipo de EI con enfoque económico que favoreciera a las grandes corporaciones. BAT parece haberse mostrado favorable a este tipo de EI ya que contribuía a promover sus intereses europeos al establecer normas básicas para la elaboración de políticas que: (i) proporcionarían un marco económico para la evaluación del conjunto de las decisiones políticas, dando prioridad implícita a los costes para las empresas; (ii) garantizarían la pronta implicación de las empresas en debates políticos; (iii) concederían al sector empresarial una ventaja continua sobre otros actores al hacer que los responsables políticos se vuelvan cada vez más dependientes de la información facilitada por el mismo; (iv) les permitirían a las empresas disponer de medios persuasivos para cuestionar legislaciones potenciales y existentes. Los datos revelan que una campaña de cabildeo que siguió, en gran parte liderada por BAT, contribuyó a asegurar la incorporación de modificaciones vinculantes al Tratado de la UE, mediante el Tratado de Ámsterdam, lo que requirió que los responsables políticos de la UE minimizaran las cargas legales que pesan sobre las empresas. Más adelante, todo el empeño se encaminó en asegurarse que dichas modificaciones a los Tratados se tradujeran en la aplicación de un tipo de EI con enfoque económico (análisis coste-beneficio - ACB) en los procedimientos de elaboración de políticas de la UE. Tanto la industria del tabaco como la industria química han recurrido desde entonces a dicho tipo de EI con la clara intención de socavar aspectos claves de políticas europeas diseñadas para proteger la salud pública.

**Conclusiones:** Nuestros resultados indican que BAT y sus aliados en el sector empresarial han cambiado en profundidad el modo en que vienen elaboradas todas las políticas de la UE, al hacer obligatorio el uso de EI con enfoque económico. El hecho de que hoy todas las decisiones políticas importantes de la UE queden obligadas a someterse a una evaluación que se inscribe dentro de este marco basado en el modelo ACB confiere una ventaja única a las grandes empresas. Lo dicho aumenta la probabilidad de que la UE desarrolle políticas que favorezcan los intereses de las grandes empresas, incluidas las que fabrican productos perjudiciales para la salud, más que el interés de sus ciudadanos. Dado que la comunidad de salud pública, orientándose hacia la EI en el ámbito de la salud, ha acogido en gran medida con satisfacción el creciente interés político por la EI, parece necesario que se examinen urgentemente los diferentes modos en que se puede utilizar la EI para socavar, así como para apoyar, políticas de salud pública eficaces.
